# Supplementary material for: An accuracy-aware extension to lrp-based pruning for CNNs to prevent cascading accuracy degradation in data-scarce transfer learning
Source: Sci Rep. 2026 Apr 13;16:17202. doi: 10.1038/s41598-026-47992-8 (PMC13233831; doi:10.1038/s41598-026-47992-8)
Supplement: Supplementary file 1 — Supplementary Information. [file 41598_2026_47992_MOESM1_ESM.pdf]

Supplementary Information: Pseudo-code of DPX-SD

Algorithm 1 presents the pseudo-code for the proposed method described in Sec. 3.2. The auxiliary functions appearing within the pseudo-code are described in the "Details of Auxiliary Functions" section.

Algorithm 1 DPX-SD: Dynamic Pruning by eXplain for Scarce Data

Input: CNN model $\mathbf{M}$, filter total number F_{num}, reference images $\mathbf{X}_r$, reference labels $\mathbf{Y}_r$, test images $\mathbf{X}_t$, test labels $\mathbf{Y}_t$, pruning step P_{step}%, max total skips T_{max}, max pruning rate P_{max}%

$P = 0$, $P'_{step} = P_{step}$ ▷ Initial pruning rate and step
$A = \text{EvaluateFunction}(\mathbf{M}, \mathbf{X}_r, \mathbf{Y}_r)$ ▷ Evaluate A
while $P < P_{max}$ **:**
  $\mathbf{S} = [0, 0, \ldots, 0]$ ▷ Initialize a vector of the sum of relevance for each filter (length F_{num})
  $P' = P + P'_{step}$ ▷ Temporary update of pruning rate
  for $\mathbf{x}_r$ in $\mathbf{X}_r$ **:**
    $\mathbf{R}_{\mathbf{x}_r} = \text{LRP}(\mathbf{M}, \mathbf{x}_r)$, $\mathbf{S} = \mathbf{S} + \mathbf{R}_{\mathbf{x}_r}$ ▷ Step 1 & 2: Get and sum relevance
  $\mathbf{M}' = \text{FilterPruner}(\mathbf{M}, \mathbf{S}, P')$ ▷ Step 3 : Remove P'% lowest relevance filter
  $A' = \text{EvaluateFunction}(\mathbf{M}', \mathbf{X}_r, \mathbf{Y}_r)$
  if $0 < (A - A')$ **:** ▷ Start **"Change of pruning rate"**(See Sec. 3.2.1)
    if $1 < \text{INT}(P'_{step} F_{num})$ **:** ▷ $\text{INT}(P'_{step} F_{num})$ is number of filters to be pruned
      $P'_{step} = P'_{step}/2$ ▷ Reduce additional pruning rate by half
    else: ▷ Start **"Change of pruning order"**(See Sec. 3.2.2)
      $T = 0$, $\mathbf{D} = \{\}$ ▷ Initialize total skip count and save dictionary
      while $0 < (A - A')$ **:**
        if $T < T_{max}$ **:**
          $\mathbf{S}' = \text{BoostLowRelevance}(\mathbf{S}, T)$ ▷ Maximize lower T relevances
          $\mathbf{M}' = \text{FilterPruner}(\mathbf{M}, \mathbf{S}', P')$, $A' = \text{EvaluateFunction}(\mathbf{M}', \mathbf{X}_r, \mathbf{Y}_r)$
          $\mathbf{D}[A'] = M'$ ▷ Register M' in D with A' as the key
          $T = T + 1$ ▷ Count up T
        else:
          $\mathbf{M}', A' = \text{BestModelChoice}(D)$ ▷ Choose best A' model from D
          break
  $\mathbf{M} = \mathbf{M}'$, $A = A'$, $P = P'$, $P'_{step} = P_{step}$ ▷ Update of various variables
  $Acc = \text{TestFunction}(\mathbf{M}, \mathbf{X}_t, \mathbf{Y}_t)$ ▷ Test pruned model and check accuracy
  print(Acc)
return M

Details of Auxiliary Functions

FilterPruner($\mathbf{M}, \mathbf{S}, \mathbf{P}'$) This function takes the model $\mathbf{M}$ and the sum of relevance vectors $\mathbf{S}$ for all filters as input, and generates a new model $\mathbf{M}'$ by removing the bottom P'% of filters with the lowest relevance scores.

BoostLowRelevance($\mathbf{S}, T$) This function significantly increases the relevance scores of the T filters with the lowest values in the sum of relevance vectors $\mathbf{S}$, thereby temporarily excluding them from pruning. This process is utilized in the "Change of pruning order" described in Sec.3.2.2.

BestModelChoice(D) This function receives a dictionary structure $\mathbf{D}$, which stores the evaluation value A' as keys and the pruned models as values, as input, and selects the model $\mathbf{M}'$ that exhibits the highest evaluation value from among them.

EvaluateFunction(M, $\mathbf{X}_r, \mathbf{Y}_r$) This function computes the harmonic mean accuracy A across all classes using the reference dataset $(\mathbf{X}_r, \mathbf{Y}_r)$, as defined in Eq. 3.

TestFunction(M, $\mathbf{X}_t, \mathbf{Y}_t$) This function evaluates the generalization performance of the pruned model $\mathbf{M}$ using the independent test dataset $(\mathbf{X}_t, \mathbf{Y}_t)$. It calculates and returns the standard top-1 classification accuracy. This value is used to report the final performance of the model at each pruning step (e.g., for plotting accuracy curves).
